# Neonatal arterial iliac thrombosis in type-I protein C deficiency: a case report

**DOI:** 10.1186/1824-7288-36-23

**Published:** 2010-03-08

**Authors:** Elisabetta Tridapalli, Marcello Stella, Maria G Capretti, Giacomo Faldella

**Affiliations:** 1 Department of Preventive Paediatrics and Neonatology - University of Bologna, Policlinico S Orsola- Malpighi, Via Massarenti 11 40138 Bologna, Italy; 2 Paediatric and Neonatal Intensive Care Unit, Ospedale M. Bufalini, Viale Ghirotti 286, 47023 Cesena, Italy

## Abstract

A male infant born by caesarean section at 38 weeks of gestational age (B.W. 4055 g; Apgar 9-10), in the first two hours of life his right leg became hypovascularizated.

Normal values of leukocities, red cells, haematocrit, hemoglobin, platelets. C-Reactive Protein negative. Electrolytes and coagulation tests were normal. Normal vitamin K coagulation proteins levels. Serological tests for TORCH (IgM) and Parvovirus (IgG and IgM) were negative.

Sonography showed a reduced blood flow in the iliac artery and reported a 1 cm long vessel thrombosis.

From 8 hours of life we administred an intravenous infusion of unfractionated heparin (UFH) 75 UI/Kg for the first 10 minutes then 28 UI/Kg/h.

On the 2^nd ^day tests were performed to assess absence of inhibiting-clot factors. The dosage of homocysteine, protein S and antithrombin was normal. FV Leiden and antiphospholipid antibodies were negative. The mapping of G20210A prothrombin's gene resulted normal, whereas the concentration of Protein C was lower than normal: activity 46% (68-150%), antigen 35% (70-150%).

The same deficiency was also found in the father. The mother showed normal concentrations. No episodies of thrombosis events were documentated in the family.

The intravenous unfractionated heparin (UFH) therapy was replaced after 64 hours by subcutaneous nadroparin 600 UI twice/day, which was stopped 5 days later when the vessel sonografic images were completely normal. During the hospitalization the infant didn't show bleeding.

The child was followed-up yearly until 4 years of age: he was well and had a normal body and mental development.

The final diagnosis is likely to be of a permanent protein C deficiency in heterozygous form. Our case is interesting because the first manifestation was an important thrombosis of large vessel that occurred within a few hours of life in absence of perinatal risk factors, as if it was a homozygous disease, but the patient had a heterozygotic form. In literature few cases are reported of heterozygous forms that became symptomatic, but only in old age.

After a severe first manifestation, a normal and asymptomatic development is uncommon without new thrombotic episodes. In our patient the neonatal thrombosis was the sole event in his life.

## Introduction

In the neonatal period thrombosis is a rare event. In two registers from Canada and Germany [1,2] the incidence of this pathology was respectly 2.4/1000 admission to NICU and 5.1/100000 births. The main causes are the presence of central line, asphyxia, septicaemia, dehydration and maternal diabetes.

Anormalities of antithrombin and the protein C system have been documented in association with spontaneous neonatal thrombotic problems.

Protein C deficiency is a genetic trait that predisposes to the formation of venous or arterial clots.

There are two kinds of Protein C deficiency: type I and type II. Type I deficiency results from an inadequate amount of protein C, that functions normally, but the quantity of protein C present is insufficient to control the coagulation cascade. Type II deficiency is characterized by defective protein C molecules: the protein C level is normal, but it's unable to interact with the other molecules involved in coagulation.

## Case Report

A male infant born by caesarean section at 38 weeks of gestational age and with a birth weight of 4055 g (Apgar Score: 9 at 1' and 10 at 5'), was referred to our NICU because in the first two hours of life his right leg became pale, hypothermic and cyanotic and oxygen saturation was lower than in the other limbs (70-80% vs. 98-100%).

Maternal vaginal scrub was negative for Agalactie group B Streptococcus and maternal screening for the common infections had negative results (HBV negative, HIV negative, CMV immune, Rubella virus immune, Toxoplasma Gondii not immune, Syphilis negative). No desease was referred during the pregnagny.

At birth a single intramuscular dose of vitamin K was administered as antihaemorrhagic prophylaxis on the left thigh.

The hypovascularization of his right leg was confirmed, even though he wasn't in immediate danger when he was admitted.

Laboratory studies revealed normal values of leukocities, red cells, haematocrit, hemoglobin, platelets. (White Blood Cells 13.080/mmc: N 68.5%, L 24.7%, M 3.6%, E 1.2%, B 0.9%; Red Cells 5.660.000/mmc, Hb 19.1 g/dl, Hct 56.2%, PLT 158.000/mmc). C-Reactive Protein negative. Electrolytes and coagulation tests were normal: aPTT-ratio 1.09 range (0.8-1.20); PT% 70% (70-100%); INR 1.27 (0.92-1.30). Our laboratory found normal vitamin K coagulation proteins levels (FII, FV, FVII, FVIII, FIX, FX, FXI, FXII). Serological tests for TORCH (IgM) and Parvovirus (IgG and IgM) were negative.

Sonography showed a reduced blood flow in the iliac artery and reported a 1 cm long vessel thrombosis. His heart and other arteries appeared to be normal. Cerebral and renal ultrasound findings were normal.

From 8 hours of life we administred an intravenous infusion of unfractionated heparin (UFH) 75 UI/Kg for the first 10 minutes then 28 UI/Kg/h.

On the 2^nd ^day tests were performed to assess presence/absence of inhibiting-clot factors. The dosage of homocysteine, protein S and antithrombin was normal. FV Leiden and antiphospholipid antibodies were negative. The mapping of G20210A prothrombin's gene resulted normal, whereas the concentration of Protein C was lower than normal: activity 46% (normal range 68-150%), antigen 35% (70-150%).

The same deficiency was also found in the father: activity 61%, antigen 50%, but he was asymptomatic. The mother showed normal protein C concentrations. No episodies of thrombosis or other thromboembolic events were documentated in father's family.

The newborn showed both a clinical and sonographic gradual improvement.

The intravenous unfractionated heparin (UFH) therapy was replaced after 64 hours by subcutaneous nadroparin 600 UI twice/day, which was stopped 5 days later when the vessel sonografic images were completely normal.

The patient was discharged from the hospital after 10 days with a year long prophylactic 20 mg/day oral dose of aspirin. During the hospitalization the infant didn't show bleeding, the cerebral sonography at the discharge resulted normal. The child was followed-up yearly until 4 years of age: he was well and had a normal body and mental development. Therapy with plasma or protein C was never necessary.

During the observational period the concentration of Protein C was confirmed low: at 1 year of life activity was 46% and antigen was 35%; at 2 years activity 47% and antigen 48%, at 3 years activity 52% and antigen 50%, at 4 years activity 40% and antigen 34%.

## Discussion

The protein C levels gradually improve during life [3]. Protein C concentration is 15% of the adult level in the infant, 35% in the premature newborn, 80% in the adolescent and it improves by 4% every 10 years [4,5].

Protein C deficiency is present in approximately 0.2% of the population. This rate includes asymptomatic people and patients with severe thrombotic disease. Symptoms depend on the genetic mutation on the chromosome 2 q13-q14. People with heterozygous form can usually live all their life without clinical problems. The classical neonatal manifestation of homozygous protein C deficiency is a severe form of thrombosis of the large vessels or a purpura fulminans that occurs within a few hours or days of life, causing tissue necrosis and gangrenous and disseminated intravascular coagulation [3,6,7].

Treatment of a patient with protein C deficiency depends on the individual patient's risk of thromboembolic disease.

In patients with homozygous form the risk of death from thrombosis is imminent. As a result, treatment is based on providing a source of Protein C [3]. This can be done with fresh frozen plasma (FFP) or with human plasma protein C concentrate. At this time there are still no studies that compare the use of the protein C concentrates versus FFP in severe protein C deficiency related thrombosis.

The treatment of deep thrombosis is commonly defined in a bolus dose of unfractionated heparin (UFH) from 75 to 100 U/kg and a maintenance dose of 28 U/Kg/h for newborns or 20 U/Kg/h for children >1 year of age [8]. The few studies on UFH in newborns show that the clearance is faster than that for older children due to a larger volume of distribution [9,10]. Also pharmacokinetic research shows the same different UFH clearance between piglets and adult pigs [11].

An alternative recommendation is the only supportive care, waiting for anticoagulation if radiologic monitoring show an extension of the thrombosis [8]. Central venous line should be removed if possible.

In our case report the final diagnosis is likely to be of a permanent protein C deficiency in heterozygous form, especially considering the patient's clinical history and father's protein C levels. Our case is interesting because the first manifestation was an important thrombosis of large vessel that occurred within a few hours of life in absence of perinatal risk factors, as if it was a homozygous disease, but the patient had a heterozygotic form. In literature [6,8,11-14], thought the clinical pattern of heterozygous disease is commonly described as very variable, few cases are reported of heterozygous forms that became symptomatic, but only in old age.

After a severe first manifestation of the thrombosis a normal and asymptomatic development is uncommon without new thrombotic episodes. In our patient the neonatal thrombosis was the sole event in his life. The child has grown well until now without physical or mental problems.

As these patients are often asymptomatic, to perform coagulation tests and the dosage of protein C on parents is the only way to identify newborns with a high risk of fatal thrombotic events. Further epidemiological studies are necessary to asses the real benefit/cost ratio of a parental prenatal screening to diagnose neonatal cases and to begin therapy sooner.

## Competing interests

The authors declare that they have no competing interests.

## Authors' contributions

ET took care of patient during the hospitalization and follow up of the patient. MS provided translating and sending of the manuscript. All authors read and approved the final manuscript. 

## Consent

Written informed consent was obtained from the patient for publication of this case report and accompanying images. A copy of the written consent is available for review by the Editor-in-Chief of this journal.
